# Effect of Digital Exercise Therapy on the Pain and Physical Function of Patients With Osteoarthritis: Systematic Review and Meta-Analysis

**DOI:** 10.2196/66037

**Published:** 2025-04-25

**Authors:** Jing Long, Jikai You, Yanan Yang

**Affiliations:** 1 School of Physical Education Jiangxi Normal University Nanchang China; 2 Department of Key Lab of Aquatic Sports Training Monitoring and Intervention of General Administration of Sport of China Jiangxi Normal University Nanchang China

**Keywords:** exercise program, telerehabilitation, rehabilitation, digital care, physical activity, health app, physical therapy, systematic review, meta-analysis

## Abstract

**Background:**

Osteoarthritis (OA) is a chronic degenerative bone and joint disease that significantly impacts patients’ quality of life and mental health, while also imposing a substantial economic burden on society. However, access to rehabilitation for patients with OA is challenging upon hospital discharge. Digital exercise therapy represents a promising telemedicine strategy for enhancing the management of OA, but its effect on OA is not yet clear.

**Objective:**

This study aimed to systematically evaluate the therapeutic effects of digital exercise therapy on pain and physical function in patients with OA.

**Methods:**

Databases including PubMed, Embase, Cochrane Library, Web of Science, and SPORTDiscus were searched for randomized controlled trials on using digital exercise therapy for OA until October 25, 2023. The primary outcomes included the measures of pain scores or physical function scores immediately after the intervention and at full follow-up. The risk of bias was evaluated using the Physiotherapy Evidence Database scale. Relevant data were extracted, and a meta-analysis was performed using RevMan5.3 software (Cochrane Collaboration).

**Results:**

A total of 9 studies with 1604 patients were included in the final meta-analysis. Compared with the conventional treatment group, digital exercise therapy significantly reduced numerical rating scale pain scores (mean difference [MD]=–1.07, 95% CI –1.35 to –0.78; *P*<.001) and Western Ontario and McMaster Universities Osteoarthritis Index physical function scores (MD=–2.39, 95% CI –3.68 to –1.10; *P*<.001) in patients with OA immediately after the intervention. However, follow-up results revealed no statistically significant difference in numerical rating scale pain scores (MD=–0.20, 95% CI –0.59 to 0.20; *P*=.34), while Western Ontario and McMaster Universities Osteoarthritis Index physical function scores showed a significant improvement in the digital exercise therapy group compared with the control group (MD=–1.89, 95% CI –3.52 to –0.26; *P*=.02). These findings suggest that digital exercise therapy provides immediate benefits for both pain and physical function in patients with OA, with sustained improvements in physical function observed during follow-up, though pain relief may not persist long term.

**Conclusions:**

Digital exercise therapy can alleviate the pain and improve the physical function in patients with OA and can be used as an auxiliary means in the rehabilitation treatment of OA. It provides great convenience for patients with OA who need long-term treatment, allowing them to exercise at home for rehabilitation training.

**Trial Registration:**

PROSPERO CRD42023484819; https://www.crd.york.ac.uk/PROSPERO/view/CRD42023484819

## Introduction

Osteoarthritis (OA) is the most common joint disease, characterized by changes in cartilage, bone hypertrophy, and the formation of bone spurs, affecting over 7% of the global population [1,2]. Recent estimates from the Global Burden of Diseases, Injuries, and Risk Factors Study indicate that the age-standardized prevalence and incidence rates of symptomatic, radiographically confirmed OA have increased by 9.3% (95% uncertainty interval 8%-10.7%) and 8.2% (95% uncertainty interval 7.1%-9.4%), respectively, between 1990 and 2017 [3,4]. In the 2019 Global Burden of Musculoskeletal Diseases survey, OA accounted for 20.1% of the demand for musculoskeletal rehabilitation, imposing a substantial economic and public health care burden on individuals, families, and society [5,6]. As a degenerative musculoskeletal disease, the incidence of OA increases with advancing age, exacerbating related societal health care challenges and necessitating comprehensive therapeutic interventions.

The primary goals within clinical treatment paradigms for OA include pain relief, deceleration of disease progression, and enhancement of joint function [7]. Exercise is recommended by current clinical guidelines as an effective treatment for patients with OA, complementing surgical interventions, pharmacological treatments, and physical therapy [5]. According to the Osteoarthritis Research Society International and the American College of Rheumatology guidelines, irrespective of comorbid conditions, dietary and weight management, consistent and enduring exercise regimens, and mind-body practices (eg, tai chi and yoga) should be considered integral therapeutic approaches for OA [6,8]. Hurley et al [9] demonstrated that exercise enhances physical function, alleviates depression, and reduces pain in patients with OA. Gay et al [10] revealed that patients with early-stage and mild OA, especially those who opted against surgical intervention, experienced substantial relief from clinically significant pain through participation in a self-management program. However, many patients face challenges in accessing rehabilitation services, particularly when transitioning from hospital care or when managing their condition in community settings [11]. Therefore, it is necessary to develop new therapeutic approaches to address this disease.

Despite the benefits of exercise therapy, access to conventional programs can be limited due to the requirement for supervised clinic sessions, time commitments, and associated costs [12]. Additionally, the treatment of these disorders often necessitates long-term care and follow-up, posing further challenges. Consequently, these programs can become inaccessible or inconvenient for many individuals. With the rapid development of internet technology, the World Health Organization (WHO) has recognized the potential of mobile technology to transform medical services [13]. Increasingly, individuals are accessing health and exercise services and resources through internet-based platforms. During the COVID-19 pandemic, home telerehabilitation became a widely used strategy for OA rehabilitation, with patients guided remotely by therapists using telecommunication technology [13-15]. Internet-based interventions offer the potential to reach a broad audience at minimal or no cost, irrespective of geographical location, enabling exercise to be performed from the comfort and convenience of home, thereby mitigating some of the barriers to exercise reported in the OA population [16].

The rise of telemedicine has increased patient access to real-time communication with professional physicians or therapists [17]. Digital exercise therapy has been developed within the fields of physical medicine and rehabilitation to provide continuous rehabilitation services for patients with disabilities [18]. Digital exercise therapy, integrating internet technologies with physical medicine and exercise, could significantly improve patient access to professional physicians or therapists, including those in remote areas [19]. However, there is a lack of high-quality evidence to confirm the clinical efficacy and superiority of digital exercise therapy. Consequently, this meta-analysis aims to systematically evaluate the efficacy of digital exercise therapy in reducing pain and improving physical function among patients with OA, thereby providing evidence-based guidance for clinicians and patients.

## Methods

### Ethical Considerations

All analyses were conducted using data from previously published studies. Therefore, no ethical approval or patient consent was required.

### Study Registration

This review was conducted in accordance with the PRISMA (Preferred Reporting Items for Systematic Reviews and Meta-Analyses) statement [20] and its associated checklist (Multimedia Appendix 1). The study was registered with PROSPERO (CRD42023484819). This systematic review was executed in strict alignment with the PROSPERO protocol, maintaining complete consistency in search strategies, inclusion and exclusion criteria, outcome definitions, and statistical approaches as originally registered.

### Literature Search Strategies

A literature search was performed until October 25, 2023, using the following databases: PubMed, Embase, Cochrane Library, Web of Science, EBSCO, and SPORTDiscus. The keywords used in the search were “osteoarthritis,” “exercise therapy,” “Web-based Intervention,” and “Self-Management.” Boolean operators “AND” and “OR” were used to combine keywords for a broader systematic search. Details of the search are presented in Multimedia Appendix 2.

### Selection Process

Two independent reviewers (JL and JY) retrieved records from 6 databases and imported them into EndNote X9 (Clarivate Analytics). Duplicate records were systematically identified and removed using EndNote X9’s deduplication function, followed by manual verification to ensure accuracy. Following duplicate removal, a manual field-based screening was conducted in EndNote X9 to exclude records violating predefined inclusion criteria. This involved filtering the “Reference Type” field to exclude conference abstracts (eg, using “Conference” as a keyword) and searching titles for keywords to exclude systematic reviews, meta-analyses, trial protocols, questionnaire studies, and pilot studies (eg, “review,” “meta-analysis,” “protocol,” “questionnaire,” and “pilot”).

The study selection process was conducted in two stages. First, titles and abstracts were screened independently by 2 reviewers (JL and JY) to exclude irrelevant studies. Second, the full texts of the remaining potentially relevant studies were reviewed independently by the same reviewers (JL and JY) to determine eligibility. Any disagreements during both stages were resolved by a third reviewer (YY). Finally, backward searches of reference lists from included studies and relevant systematic reviews were performed by the initial reviewers (JL and JY) to ensure comprehensiveness. During the initial screening, no restrictions were placed on the specific pain measurement tools used. The primary criterion for inclusion was the reporting of pain measures, physical function measures, or both, regardless of the specific scale used Textbox 1.

**Inclusion and exclusion criteria.**

**Inclusion criteria**

- Randomized controlled trials evaluating the efficacy of digital exercise therapy compared with conventional treatment groups
- Study populations consisting of adults diagnosed with osteoarthritis
- Participants in the intervention group received digital exercise therapy
- The conventional treatment groups could be of any type (eg, waiting list, usual care, or minimal interventions) as long as participants did not use apps providing personalized video exercises to support rehabilitation
- Studies reported at least one of the following clinical outcomes: pain measures (preferably using the numeric rating scale) or physical function scores
- Studies published in English

**Exclusion criteria**

- Review articles, conference proceedings, and animal studies
- Nonrandomized controlled trials, republished papers, or papers with poor-quality evaluation
- Literature not in English
- Full text not available
- Outcome indicators did not meet the requirements, or data could not be extracted

### Outcome Measures

The primary outcomes evaluated were pain intensity and physical function. Pain intensity was measured using the numeric rating scale (NRS), a widely used tool for pain assessment. The NRS is an 11-point, unidimensional, horizontal scale where “0” indicates “no pain” and “10” indicates “worst possible pain.” Patients rate their pain by choosing a number that best reflects their current pain level. The NRS is easy to administer and score and is sensitive to changes in pain levels, making it popular in both clinical and research settings [21,22]. Physical function was measured using the Western Ontario and McMaster Universities Osteoarthritis Index (WOMAC), a standardized, self-administered questionnaire widely used to assess the impact of OA on patients’ lives. The WOMAC consists of 24 items divided into three subscales: pain (5 items), stiffness (2 items), and physical function (17 items). Each item is scored on a 5-point Likert scale, with higher scores indicating greater severity of symptoms and functional limitations [23,24].

### Data Extraction

Two reviewers (JL and JY) independently extracted data using a standardized form, including study characteristics (design and sample size), participant demographics, intervention and control details, outcome measures (mean and SD for the NRS and WOMAC immediately after the intervention and at follow-up), and discrepancies were resolved through discussion. Corresponding authors were contacted for missing data. Upon reviewing the data, we found that most studies included in our analysis used the NRS to measure pain. However, one study [25] reported pain using the WOMAC pain score. To maintain consistency in our meta-analysis, we decided to include only the NRS data. This decision was made to ensure uniformity in the quantitative synthesis of pain outcomes across studies. The study using the WOMAC pain score was still included in the overall review but was excluded from the meta-analysis of pain outcomes.

### Assessment of Quality Evidence

Quality assessments were performed with the Physiotherapy Evidence Database (PEDro) scale [26], which is based on the Delphi List criteria and is considered valid and reliable to evaluate the methodological quality of included studies. All included trial reports were checked in the PEDro database to confirm their PEDro scale score. Considering that criterion 1 was not used to calculate the score, the sum of the other criteria could have a maximum of 10 points. Trials with a score ≥6 points were classified as “good,” whereas those with a score ≤5 points were classified as “poor” and excluded from the analysis. If a PEDro score was not available in the database, 2 authors independently assessed study quality using the PEDro scale and its associated notes. Any disagreements were resolved through discussion until a consensus was reached [27].

### Statistical Analysis

Based on the availability of the reported data, the primary outcomes selected for meta-analysis were the NRS score and the WOMAC score. These outcomes were analyzed immediately after the intervention and at follow-up). As the data were continuous, the mean and SD were used to calculate the mean difference (MD) with a 95% CI. The primary meta-analysis was performed using Review Manager 5.4 software (The Nordic Cochrane Centre). Fixed-effects statistical models were applied as the default in the meta-analysis. If heterogeneity was found, random-effects models were applied. The significance level was set at *P*<.05, and results were presented using forest plots [28-30].

## Results

### Search and Selection

**Figure 1:**
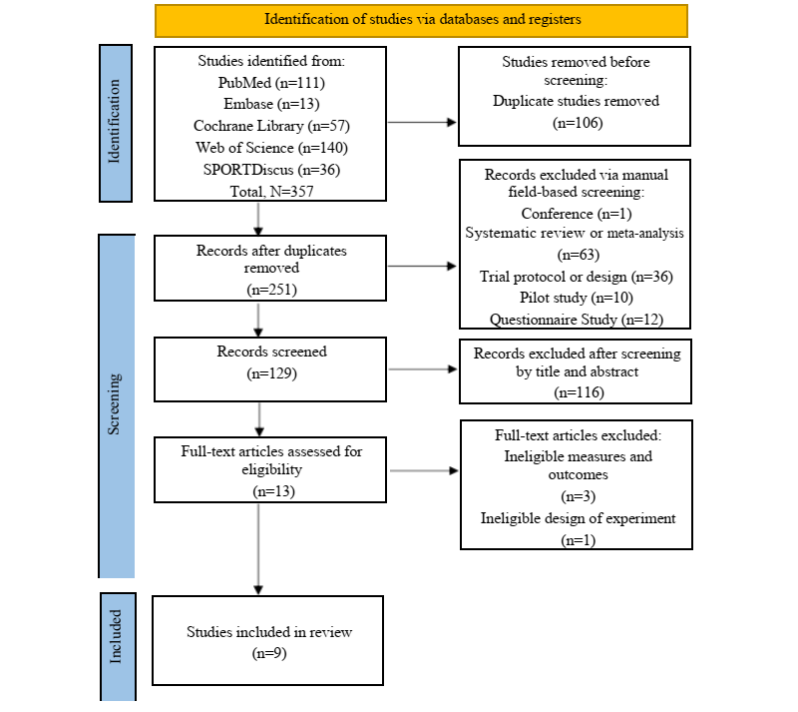
PRISMA (Preferred Reporting Items for Systematic Reviews and Meta-Analyses) flow diagram of the review.

Figure 1 depicts the study selection process. Initially, 357 publications were retrieved through electronic database searches. After the removal of 106 duplicates, 251 articles were assessed using manual field-based screening, leading to the exclusion of 122 articles for various reasons, including conference papers, systematic reviews or meta-analyses, trial protocols or designs, pilot studies, and questionnaire-based studies. Based on predefined inclusion and exclusion criteria, the titles and abstracts of 129 articles were further evaluated. Among these, 116 articles were excluded as they did not investigate or compare the combination of digital exercise therapy with conventional treatment in patients with OA. After a detailed review of the remaining 13 full-text articles, 4 additional articles were excluded for the following reasons: ineligible outcome measures, irrelevant outcomes, or an ineligible study design. Consequently, only 9 articles were deemed suitable for further review and analysis. This meticulous selection process ensured that only relevant and high-quality studies were included in the subsequent analysis, thereby enhancing the validity of the findings [18,25,31-37].

### Basic Characteristics of the Included Studies

All 9 randomized controlled trials (RCTs) included in the meta-analysis were written in English between 2016 and 2023 [18,25,31-37]. A total of 1604 participants were enrolled, with 798 (49.7%) in the digital exercise therapy group and 806 (50.2%) in the conventional treatment group. Detailed characteristics of the eligible studies are presented in Table 1.

All 9 studies involved adult participants, with 4 studies including participants aged ≥45 years [18,31,33,36], 3 studies including participants aged ≥50 years [25,32,35], 1 study including participants aged >18 years [34], and 1 study including participants aged 40-80 years [37]. Overall, 7 studies focused specifically on knee OA [18,25,31-33,35,36], 1 study focused on knee OA or hip OA [37], and 1 study focused on hand OA [34]. The inclusion of participants from different age groups and the focus on various types of OA contributed to the diversity and representativeness of the included studies.

**Table 1:** Participant characteristics of included studies.

Study (participants)			Female, n (%)		Age (years), mean (SD)
**Bennell et al [32], 2016 (N=168)**					
	Experimental group^a^ (n=84)	57 (68)		61.1 (6.9)	
	Control group^b^ (n=84)	49 (58)		63.4 (7.8)	
**Bennell et al [18], 2022 (N=212)**					
	Experimental group (n=107)	70 (65)		62.8 (8.2)	
	Control group (n=105)	78 (74)		61.8 (7.2)	
**Rodríguez Sánchez-Laulhé et al [34], 2022 (N=144)**					
	Experimental group (n=66)	25 (73)		62.2 (8.8)	
	Control group (n=78)	25 (62)		64.3 (7.7)	
**Nelligan et al [33], 2021 (N=206)**					
	Experimental group (n=103)	60 (58)		60.3 (8.2)	
	Control group (n=103)	66 (64)		59.0 (8.5)	
**Hinman et al [31], 2019 (N=175)**					
	Experimental group (n=87)	55 (63)		62.4 (9.1)	
	Control group (n=88)	55 (63)		62.5 (8.1)	
**Allen et al [25], 2018 (N=282)**					
	Experimental group (n=142)	98 (69)		65.3 (11.5)	
	Control group (n=140)	100 (71)		65.7 (10.3)	
**Baker et al [35], 2020 (N=104)**					
	Experimental group (n=52)	42 (81)		65.8 (6.6)	
	Control group (n=52)	42 (83)		64.5 (8.3)	
**Gohir et al [36], 2021 (N=105)**					
	Experimental group (n=48)	34 (71)		65.2 (9.7)	
	Control group (n=57)	37 (65)		68.0 (8.6)	
**Kloek et al [37], 2018 (N=208)**					
	Experimental group (n=109)	74 (68)		63.8 (8.5)	
	Control group (n=99)	67 (68)		62.3 (8.9)	

^a^Experimental group: digital exercise therapy group.

^b^Control group: conventional treatment groups.

Table 2 summarizes the 9 included trials, each featuring unique digital exercise therapy protocols. These studies varied in intervention methods, evaluation indicators, and effects. The digital exercise therapy groups used online exercises, while the conventional groups in 6 studies lacked online support [25,32,34-37], and 3 did not include an exercise intervention [18,31,33]. Diverse therapies were used: Nelligan et al [33] offered a 24-week program via a website and SMS text messages; Rodríguez Sánchez-Laulhé et al [34] introduced a 12-week app-based program for hand OA; Hinman et al [31] added physiotherapist support into a telephone service; Bennell et al [18,32] studied internet-based yoga and telephone coaching for knee OA; Allen et al [25] developed an internet-based exercise training program with tailored exercises and progress tracking; Baker et al [35] created the BOOST telephone-linked communication program focusing on exercise behavior; Gohir et al [36] developed a 6-week app program for leg strength and balance; and Kloek et al [37] combined online and face-to-face sessions in the e-Exercise program (refer to Table 1 and Table 2 for further details).

**Table 2:** Intervention information of included studies.

Study	Intervention	Control	Exposure	Follow-up points
Bennell et al [32], 2016	Usual PT^a^ care+home exercise program+telephone-delivered coaching	Usual PT care+home exercise program (without app)	3 times per week, for 24 weeks	48 weeks
Nelligan et al [33], 2021	Knee strengthening exercise program+My Knee Exercise website+SMS text messages	My Knee Education website (without exercise regimen and physical activity guidance)	Any number of times, for 24 weeks	—^b^
Hinman et al [31], 2019	Home strengthening exercise program+telephone-delivered support	Telephone-delivered support (without exercise program)	3 times per week, for 24 weeks	24 weeks
Baker et al [35], 2020	Strength training+TLC^c^	Strength training+phone message reminders (without online guidance)	Any number of times, for 96 weeks	—
Bennell et al [18], 2022	Yoga+self-directed exercise+website	Self-directed exercise+website (without yoga)	3 times per week, for 12 weeks	12 weeks
Allen et al [25], 2018	Exercise+IBET^d^ program	Exercise (without IBET program)	3 times per week, for 16 weeks	32 weeks
Rodríguez Sánchez-Laulhé et al [34], 2022	Home exercise program+CareHand (Healthinn) mobile app	Usual care (without CareHand mobile app)	4 times per week, for 12 weeks	12 weeks
Gohir et al [36], 2021	Exercise+self-management programs+smartphone app	Usual care+exercise (without app)	Any number of times, for 6 weeks	—
Kloek et al [37], 2018	Usual PT care+e-Exercise app	Usual PT care (without app)	3 times per week, for 12 weeks	36 weeks

^a^PT: physical therapy.

^b^Not available.

^c^TLC: telephone-linked communication program.

^d^IBET: internet-based exercise training.

Table 3 presents the effect sizes of digital exercise therapy interventions for both immediate and follow-up findings, categorized by digital resource type (phone interventions, app-based interventions, and website-based interventions). A total of 4 studies [31-33,35] used phone interventions (SMS text messages), showing mean effect sizes of –0.44 (95% CI –0.75 to –0.14) for NRS pain and –0.24 (95% CI –0.39 to –0.09) for WOMAC physical function immediately after the intervention. During follow-up, the mean effect sizes were –0.11 (95% CI –0.43 to 0.22) for NRS pain and –0.29 (95% CI –0.62 to 0.03) for WOMAC physical function. Two studies [18,25] used websites, with mean effect sizes of –0.29 (95% CI –0.57 to –0.01) for NRS pain and –0.13 (95% CI –0.25 to –0.01) for WOMAC physical function immediately after the intervention. During follow-up, the mean effect sizes were –0.01 (95% CI –0.09 to 0.08) for NRS pain and –0.11 (95% CI –0.37 to 0.15) for WOMAC physical function. Three studies [34,36,37] used apps, revealing mean effect sizes of –0.43 (95% CI –0.63 to –0.23) for NRS pain and –0.47 (95% CI –0.87 to –0.07) for WOMAC physical function immediately after the intervention. During follow-up, the mean effect size was 0.04 (95% CI –0.30 to 0.38) for NRS pain.

**Table 3:** The effect sizes of digital resources in the included studies.

Digital resources and studies		Immediate findings (effect size^a^)			Follow-up findings (effect size)	
		NRS^b^, mean difference (95% CI)	WOMAC^c^, mean difference (95% CI)	NRS, mean difference (95% CI)		WOMAC, mean difference (95% CI)
**Phone**						
	Bennell et al [32], 2016	–0.31 (–0.64 to 0.02)	–0.31 (–0.64 to 0.02)	–0.18 (–0.49 to 0.12)		–0.38（−0.69 to −0.07）
	Hinman et al [31], 2019	–0.33 (–0.63 to 0.00)	–0.30 (–0.61 to –0.01)	–0.04 (–0.34 to 0.25)		–0.23 (–0.52 to 0.07)
	Nelligan et al [33], 2021	–0.65 (–0.93 to –0.38)	–0.31 (–0.60 to 0.03)	—^d^		—
	Baker et al [35], 2020	—	–0.03 (–0.41 to 0.34)	—		—
	Average	–0.44 (–0.75 to –0.14)	–0.24 (–0.39 to –0.09)	–0.11 (–0.43 to 0.22)		–0.29 (–0.62 to 0.03)
**App**						
	Rodríguez Sánchez-Laulhé et al [34], 2022	–0.31（–0.66 to 0.05）	—	0.12 (–0.24 to 0.48)		—
	Gohir et al [36], 2021	–0.97 (–1.37 to –0.57)	–0.47 (–0.87 to –0.07)	—		—
	Kloek et al [37], 2018	–0.21 (–0.51 to –0.01)	—	–0.03 (–0.34 to 0.27)		—
	Average	–0.43 (–0.63 to –0.23)	–0.47 (–0.87 to –0.07)	0.04 (–0.30 to 0.38)		—
**Website**						
	Bennell et al [18], 2022	–0.29 (–0.57 to –0.01)	–0.32 (–0.54 to –0.10)	–0.01 (–0.09 to 0.08)		–0.15 (–0.42 to 0.13)
	Allen et al [25], 2018	—	0.02 (–0.18 to 0.22)	—		–0.09 (–0.32 to 0.15)
	Average	–0.29 (–0.57 to –0.01)	–0.13 (–0.25 to –0.01)	–0.01 (–0.09 to 0.08)		–0.11 (–0.37 to 0.15)

^a^Effect size: Cohen *d*.

^b^NRS: numeric rating scale.

^c^WOMAC: Western Ontario and McMaster Universities Osteoarthritis Index.

^d^Not available.

### Methodological Quality

The PEDro scale assesses the methodological quality of RCTs, with higher scores indicating better quality. All 9 studies included in the review scored high on the PEDro scales, with scores ranging from 7 to 9, indicating sufficient quality among the included studies (Table 4). An identified risk of bias across all studies was the lack of blinding for participants or therapists during the intervention, along with inadequate blinding of outcome assessors in 4 studies. Table 4 provides detailed information on PEDro scores and specific aspects of blinding for each study.

**Table 4:** Methodological quality of included studies assessed using the Physiotherapy Evidence Database scale.

Study	EC^a^	RA^b^	CA^c^	GS^d^	BP^e^	BT^f^	BA^g^	MO^h^	IT^i^	BG^j^	PM^k^	Total
Bennell et al [32], 2016	Y^l^	Y	Y	Y	Y	N^m^	Y	Y	Y	Y	Y	9
Bennell et al [18], 2022	Y	Y	N	Y	Y	N	N	Y	Y	Y	Y	8
Rodríguez Sánchez-Laulhé et al [34], 2022	Y	Y	Y	Y	Y	N	N	Y	Y	Y	Y	9
Nelligan et al [33], 2021	Y	Y	Y	Y	N	N	N	Y	Y	Y	Y	8
Hinman et al [31], 2019	Y	Y	Y	Y	N	N	N	Y	Y	Y	Y	8
Allen et al [25], 2018	Y	Y	Y	Y	N	N	N	Y	Y	Y	Y	8
Baker et al [35], 2020	Y	Y	Y	Y	Y	N	N	Y	Y	Y	Y	9
Gohir et al [36], 2021	Y	Y	Y	Y	N	N	Y	Y	Y	Y	Y	9
Kloek et al [37], 2018	Y	Y	N	Y	N	N	N	Y	Y	Y	Y	7

^a^EC: eligibility criteria.

^b^RA: randomized allocation.

^c^CA: concealed allocation.

^d^GS: groups similar at baseline.

^e^BP: blind participants.

^f^BT: blind therapists.

^g^BA: blind assessors.

^h^MO: measure of one key outcome obtained from>85% initial subjects.

^i^IT: intention-to-treat.

^j^BG: between-group comparisons

^k^PM: point measures and measures of variability.

^l^Y: yes.

^m^N: no.

### Results of the Meta-Analysis

#### Effect on Pain

**Figure 2:**
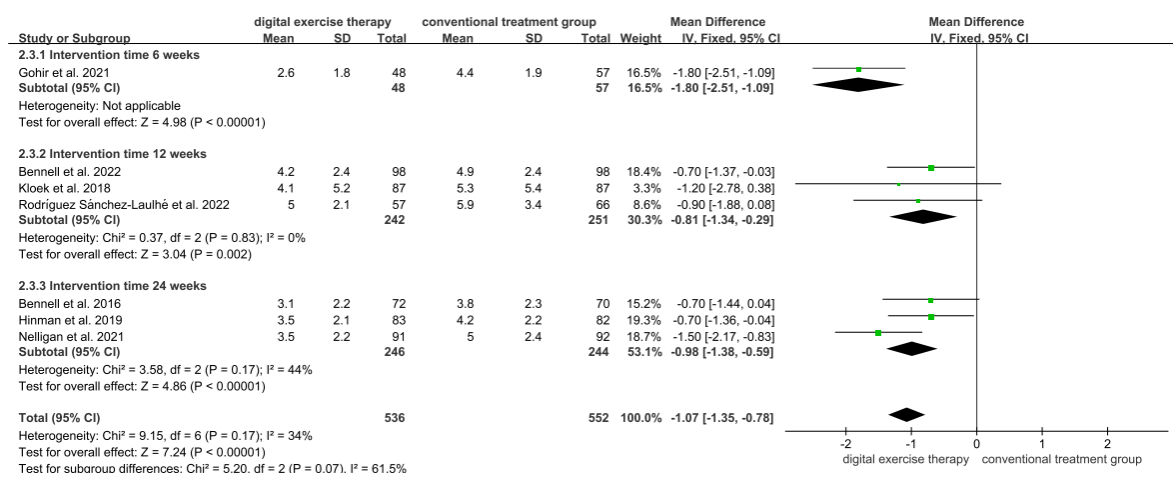
Forest plot of included studies comparing the effects of digital exercise therapy and control on the numerical rating scale for pain. IV: inverse variance.

A total of 7 studies evaluated the effect of digital exercise therapy on the pain of patients with OA using the NRS immediately after the intervention, including 1088 patients with OA—536 patients in the digital exercise therapy group and 552 patients in the conventional treatment groups (Figure 2 [18,31-34,36,37]). The meta-analysis results demonstrated that digital exercise therapy significantly reduced NRS pain scores compared with the conventional treatment group (MD=–1.07, 95% CI –1.35 to –0.78; *P*<.001). Subgroup analyses by intervention duration revealed consistent pain reduction, with significant effects at 6 weeks (MD=–1.80, 95% CI –2.51 to –1.09; *P*<.001), 12 weeks (MD=–0.81, 95% CI –1.34 to –0.29; *P*=.002), and 24 weeks (MD=–0.98, 95% CI –1.38 to –0.59; *P*<.001).

**Figure 3:**
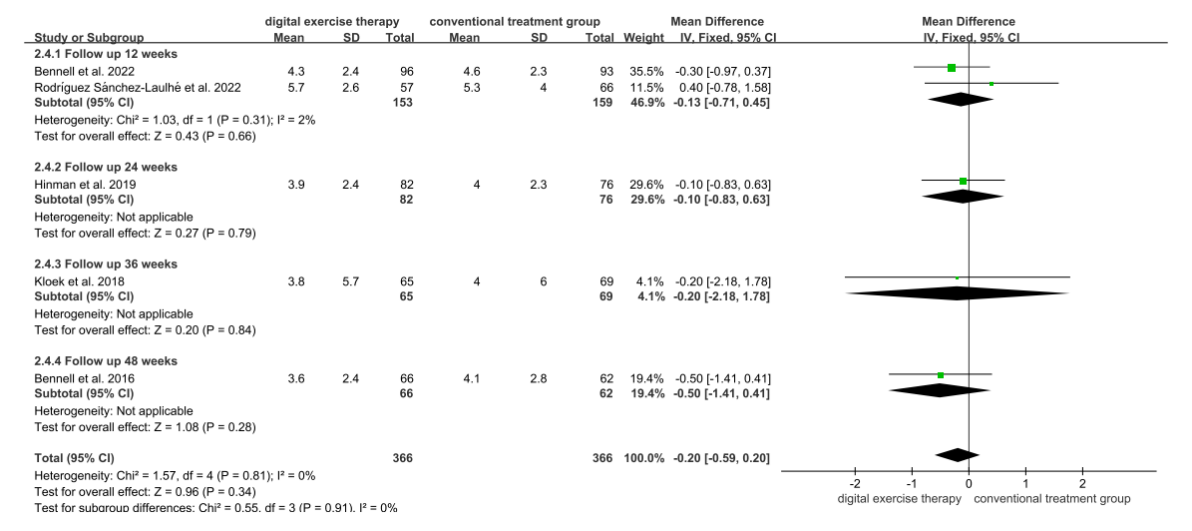
Forest plot of included studies comparing the effects of digital exercise therapy and control after follow-up on the numerical rating scale for pain. IV: inverse variance.

A total of 5 studies evaluated the effect of digital exercise therapy on the pain of patients with OA using the NRS during follow-up. In total, 732 patients were included, with 366 (50%) patients in the digital exercise therapy group and 366 (50%) patients in the conventional treatment groups (Figure 3 [18,31,32,34,37]). The follow-up results showed no significant difference in NRS pain scores between the digital exercise therapy and conventional treatment groups (MD=–0.20, 95% CI –0.59 to 0.20; *P*=.34). Subgroup analyses by follow-up duration revealed consistent pain effects, with no significant differences at 12 weeks (MD=–0.13, 95% CI –0.71 to 0.45; *P*=.66), 24 weeks (MD=–0.10, 95% CI –0.83 to 0.63; *P*=.79), 36 weeks (MD=–0.20, 95% CI –2.18 to 1.78; *P*=.84), or 48 weeks (MD=–0.50, 95% CI –1.41 to 0.41; *P*=.28).

#### Effect on Physical Function

**Figure 4:**
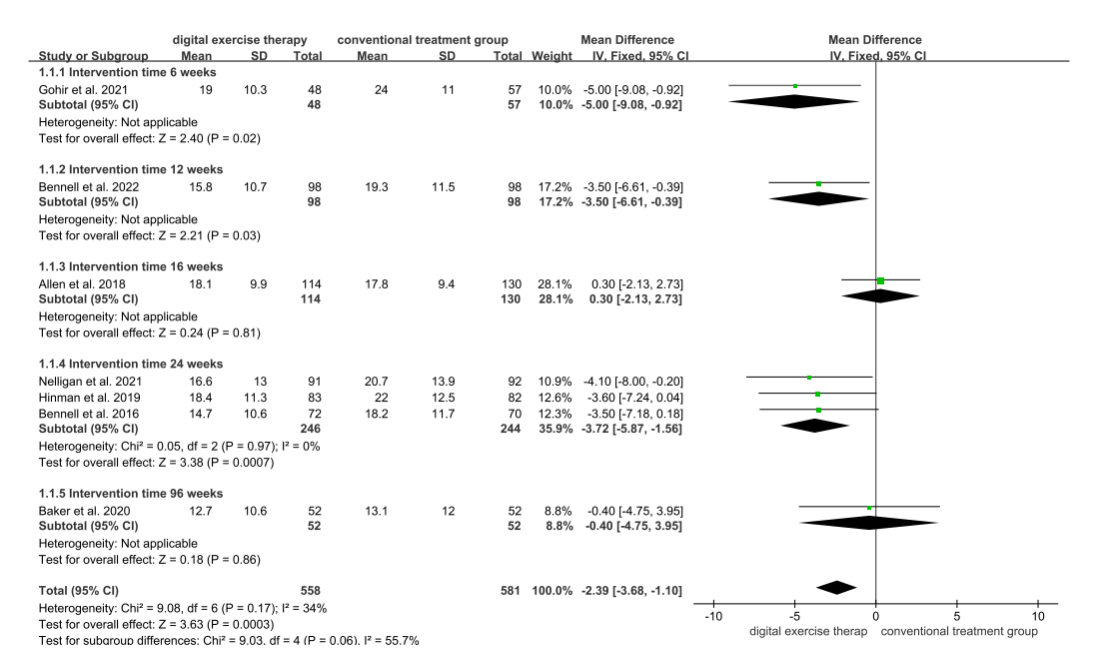
Forest plot of included studies comparing the effects of digital exercise therapy and control on physical function using the Western Ontario and McMaster Universities Osteoarthritis Index function subscale. IV: inverse variance.

A total of 7 studies evaluated the effect of digital exercise therapy on the physical function of patients with OA using WOMAC scores immediately after the intervention, involving 1139 patients, with 558 patients in the digital exercise therapy group and 581 in the conventional treatment group. The meta-analysis results demonstrated that WOMAC scores for patients in the digital exercise therapy group were significantly lower than that of the conventional treatment group (MD=–2.39, 95% CI –3.68 to –1.10; *P*<.001; Figure 4 [18,25,31-33,36]), indicating a significant improvement in physical function attributable to the digital exercise therapy intervention. Subgroup analysis revealed consistent improvements in physical function across intervention durations of 6 weeks (MD=–5.00, 95% CI –9.08 to –0.92; *P*=.02), 12 weeks (MD=–3.50, 95% CI –6.61 to –0.39; *P*=.03), and 24 weeks (MD=–3.72, 95% CI –5.87 to –1.56; *P*<.001). However, no statistically significant difference in WOMAC physical function scores was observed at both 16 weeks (MD=0.30, 95% CI –2.13 to 2.73; *P*=.81) and 96 weeks (MD=–0.40, 95% CI –4.75 to 3.95; *P*=.86).

**Figure 5:**
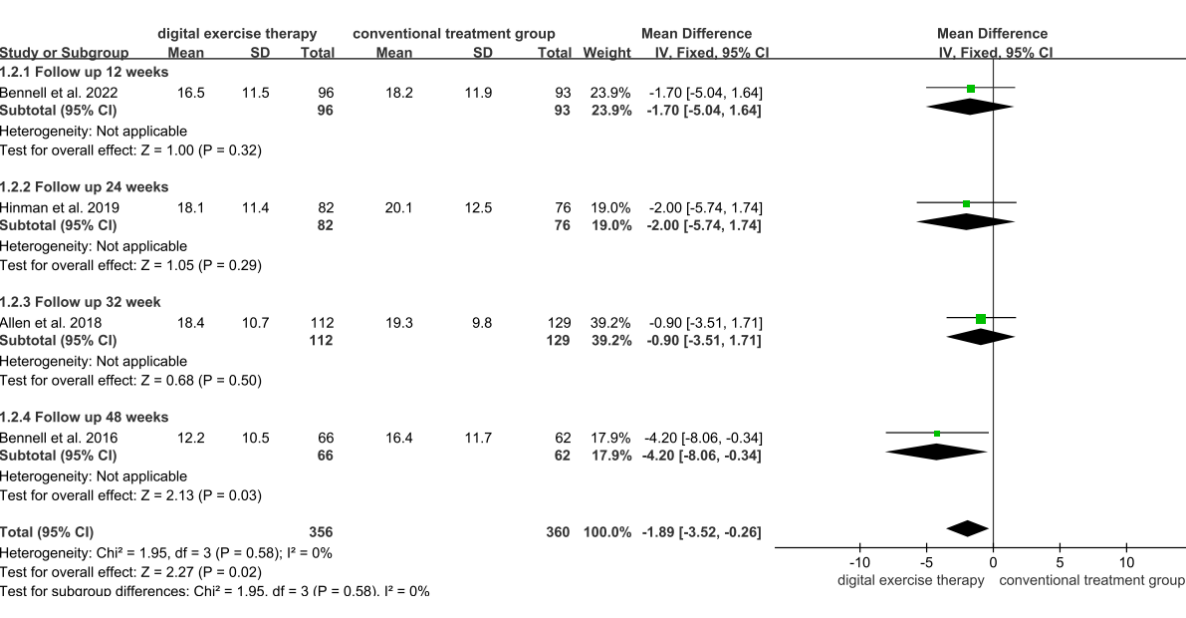
Forest plot of included studies comparing the effects of digital exercise therapy and control on physical function after follow-up using the Western Ontario and McMaster Universities Osteoarthritis Index function subscale. IV: inverse variance.

Around 4 studies evaluated the effect of digital exercise therapy on the physical function of patients with OA using WOMAC scores during follow-up, involving a total of 716 patients, with 356 patients from the digital exercise therapy group and 360 patients from the conventional treatment group (Figure 5 [18,25,31,32]). The follow-up results indicated a significant difference in WOMAC physical function scores in the digital exercise therapy group compared with the conventional treatment groups (MD=–1.89, 95% CI –3.52 to –0.26; *P*=.02). Subgroup analysis based on intervention duration revealed a statistically significant improvement in WOMAC scores during the 48 week follow-up period (MD=–4.20, 95% CI –8.06 to –0.34; *P*=.03). However, no statistically significant differences in WOMAC physical function scores were observed at 12 weeks (MD=–1.70, 95% CI –5.04 to 1.64; *P*=.32), 24 weeks (MD=–2.00, 95% CI –5.74 to 1.74; *P*=.29), and 32 weeks (MD=–0.90, 95% CI –3.51 to 1.71; *P*=.50).

## Discussion

### Principal Findings

This systematic review and meta-analysis aimed to evaluate the effectiveness of digital exercise therapy in improving pain and physical function among patients with OA. The meta-analysis included 9 studies, involving a total of 1604 patients with OA. These studies exhibited high methodological quality, as evidenced by PEDro scores exceeding 7. The results indicate that digital exercise therapy can significantly alleviate pain compared with conventional treatment immediately after the intervention, with phone-, app-, and website-based interventions demonstrating consistent pain reduction. Similarly, improvements in WOMAC physical function were observed across all modalities. Subgroup analysis revealed that the duration of the intervention did not influence the effects on pain outcomes. However, follow-up results showed no statistically significant differences in pain or physical function scores between groups, suggesting limited sustainability of benefits. Our findings align with a previous review of digital therapeutic tools for exercise programs in knee OA [38], which reported significant pain improvements in 10 of 11 studies but did not address long-term outcomes. This underscores the potential of digital modalities for short-term symptom management while highlighting the need for strategies to prolong therapeutic effects.

The loss of effect at follow-up is an important finding that warrants further exploration. Based on our analysis of the included studies, we found that changes in pain and physical function scores during follow-up were not uniform across studies. Specifically, some studies reported improvements in pain and physical function in the digital exercise therapy group compared with the control group, while others showed no significant changes or even slight worsening in some cases. Our analysis suggests that this phenomenon is multifactorial and cannot be solely attributed to increased pain in the digital exercise therapy group or decreased pain in the control group. Instead, it appears to be influenced by a combination of factors, including patient adherence to the digital exercise program [31], the duration and intensity of the intervention [25], and the natural course of OA [32,37]. Future studies should aim to investigate these factors more thoroughly to better understand the sustainability of digital exercise therapy benefits over time.

This variability suggests that the loss of effect at follow-up may be influenced by multiple factors. One possible explanation is that digital interventions primarily influence cognitive and behavioral aspects of pain management without directly addressing underlying pathological changes in OA. This hypothesis is supported by studies demonstrating that digital interventions can improve pain perception through cognitive manipulation but may not directly impact tissue damage [39,40]. Future research should focus on integrating digital exercise therapy with other therapeutic approaches, such as pharmacological treatments or lifestyle modifications, to enhance long-term efficacy. Another potential explanation for the loss of effect at follow-up is the lack of sustained engagement with digital exercise programs. Adherence to home-based exercises is a critical factor influencing long-term outcomes, and digital interventions may face challenges in maintaining participant motivation and compliance over extended periods [41-43]. Future studies should incorporate assessments of participant adherence and explore strategies to enhance long-term engagement with digital exercise therapy. Additionally, standardizing intervention protocols and incorporating personalized elements may improve the effectiveness and sustainability of digital exercise therapy.

Despite the limitations in long-term efficacy observed in this study, digital exercise therapy remains a promising approach for managing OA, particularly in settings where access to conventional rehabilitation services is limited. The COVID-19 pandemic has highlighted the potential of telehealth interventions to provide accessible and convenient care for patients with chronic conditions [13-15]. Digital exercise therapy offers flexibility and convenience, allowing patients to engage in rehabilitation from home, thereby reducing barriers related to time, transportation, and geographical constraints [44,45]. Integrating digital exercise therapy into clinical practice may enhance treatment accessibility and efficiency, particularly for patients in rural or remote areas or those with limited mobility.

However, several practical considerations must be addressed to optimize the implementation of digital exercise therapy. First, the safety and cost-effectiveness of digital interventions need to be better understood. While no increase in harm was reported in the included studies, comprehensive safety assessments and cost-effectiveness analyses are necessary to support widespread adoption [46-49]. Additionally, future research should focus on developing standardized outcome measures and incorporating multidimensional factors, such as mental health status, social support, and personal preferences, to provide a comprehensive evaluation of digital exercise therapy’s potential benefits and limitations.

### Limitations

This review has several limitations. The considerable heterogeneity in digital resource types and research designs limits the generalizability of the findings. Future studies should aim to standardize intervention protocols and outcome measures to facilitate more consistent comparisons. Additionally, the lack of long-term follow-up data and the absence of blinding methods in the included studies pose challenges in interpreting the sustained effects and safety of digital exercise therapy. Future research should prioritize long-term follow-up assessments and methodological improvements to reduce bias.

Another limitation is the relatively homogeneous sample of participants, primarily comprising middle-aged individuals from higher-income countries. Future studies should broaden the geographical and demographic scope to enhance the external validity of the findings. Furthermore, the potential impact of socioeconomic status, educational background, and digital literacy on the effectiveness of digital exercise therapy should be explored in diverse populations.

### Conclusion

Digital exercise therapy has demonstrated certain therapeutic effects in alleviating pain and improving physical function in patients with OA, and it can serve as an auxiliary means in the rehabilitation treatment of arthritis. Due to the limited sample size in this study, more high-quality, large-sample RCTs are needed in future research to further explore the efficacy of digital exercise therapy for OA.

## Appendix

Multimedia Appendix 1 PRISMA-DTA checklist.

Multimedia Appendix 2 Search strategy.

## Abbreviations

MD: mean difference
NRS: numerical rating scale
OA: osteoarthritis
PEDro: Physiotherapy Evidence Database
PRISMA: Preferred Reporting Items for Systematic Reviews and Meta-Analyses
RCT: randomized controlled trials
WHO: World Health Organization
WOMAC: Western Ontario and McMaster Universities Osteoarthritis Index

## Data Availability

All data generated or analyzed during this study are included within the published paper. The figures and associated data are publicly available through publication in an open-access journal.
