# Supplementary material for: Effect of Digital Exercise Therapy on the Pain and Physical Function of Patients With Osteoarthritis: Systematic Review and Meta-Analysis
Source: J Med Internet Res. 2025 Apr 25;27:e66037. doi: 10.2196/66037 (PMC12064974; doi:10.2196/66037)
Supplement: Multimedia Appendix 2 [file jmir_v27i1e66037_app2.pdf]

Search strategy

Search strategy in PubMed:

(((("Exercise Therapy"[Mesh]) OR (((((((((((Remedial Exercise) OR (Exercise, Remedial)) OR (Exercises, Remedial)) OR (Remedial Exercises)) OR (Therapy, Exercise)) OR (Exercise Therapies)) OR (Therapies, Exercise)) OR (Rehabilitation Exercise)) OR (Exercise, Rehabilitation)) OR (Exercises, Rehabilitation)) OR (Rehabilitation Exercises))) AND (("Osteoarthritis"[Mesh]) OR ((((((((((Osteoarthritides) OR (Osteoarthrosis)) OR (Osteoarthroses)) OR (Arthritis, Degenerative)) OR (Arthritides, Degenerative)) OR (Degenerative Arthritides)) OR (Degenerative Arthritis)) OR (Arthrosis)) OR (Arthroses)) OR (Osteoarthrosis Deformans)))) AND (((((((("Self-Management"[Mesh]) OR ("Self Management"[Title/Abstract])) OR ("Management, Self"[Title/Abstract])) OR ("self-help"[Title/Abstract])) OR ("self-administ*"[Title/Abstract])) OR ("self-guided"[Title/Abstract])) OR ("self-directed"[Title/Abstract])) OR ((("Self Care"[Mesh]) OR ("Care, Self"[Title/Abstract])) OR ("Self-Care"[Title/Abstract])))) AND ((((((((((((((((((((((((((((((((((((((("Internet-Based Intervention"[Mesh]) OR ("Internet Based Intervention*"[Title/Abstract])) OR ("Internet-Based Intervention*"[Title/Abstract])) OR ("Intervention, Internet-Based"[Title/Abstract])) OR ("Web-based Intervention*"[Title/Abstract])) OR ("Intervention, Web-based"[Title/Abstract])) OR ("Internet Intervention*"[Title/Abstract])) OR ("Intervention, Internet"[Title/Abstract])) OR ("Intervention, Online"[Title/Abstract])) OR ("social media*"[Title/Abstract]) OR ("mobile"[Title/Abstract])) OR ("web"[Title/Abstract])) OR ("application"[Title/Abstract])) OR ("App"[Title/Abstract])) OR ("WeChat"[Title/Abstract])) OR ("facebook"[Title/Abstract])) OR ("twitter"[Title/Abstract]))) OR ("Telemedicine"[Mesh]) OR ("eHealth"[Title/Abstract])) OR ("ehealth"[Title/Abstract])) OR ("e-Health"[Title/Abstract])) OR ("e-health"[Title/Abstract])) OR ("telemedicine"[Title/Abstract])) OR ("tele-medicine"[Title/Abstract])) OR ("Mobile Health"[Title/Abstract])) OR ("Health, Mobile"[Title/Abstract])) OR ("mHealth"[Title/Abstract])) OR ("m-Health"[Title/Abstract])) OR ("m-health"[Title/Abstract])) OR ("telehealth"[Title/Abstract])) OR ("tele-health"[Title/Abstract]))) OR ("Telerehabilitation"[Mesh]) OR ("Telerehabilitation*"[Title/Abstract])) OR ("Tele-rehabilitation*"[Title/Abstract])) OR ("Remote Rehabilitation*"[Title/Abstract])) OR ("Rehabilitation, Remote"[Title/Abstract])) OR ("Virtual Rehabilitation*"[Title/Abstract])) OR ("Rehabilitation, Virtual"[Title/Abstract]))) OR (((((("telecare"[Title/Abstract]) OR ("tele-care"[Title/Abstract])) OR ("telemonitoring"[Title/Abstract])) OR ("tele-monitoring"[Title/Abstract])) OR ("teleconsultation"[Title/Abstract])) OR ("tele-consultation"[Title/Abstract])))

Search strategy in Web of Science:

(((((((((TS=("Self-Management")) OR TS=("Management, Self")) OR TS=("self-help")) OR TS=("self-administ*")) OR TS=("self-guided")) OR TS=("self-directed")) OR TS=("Self Care")) OR TS=("Care, Self")) OR TS=("Self-Care")) NOT (SILOID==("PPRN")) AND ((TS=("Internet-Based Intervention")) OR TS=("Internet-Based

Intervention*")) OR TS=("Internet Based Intervention*")) OR TS=("Intervention, Internet-Based")) OR TS=("Web-based Intervention*")) OR TS=("Intervention, Web-based")) OR TS=("Internet Intervention*")) OR TS=("Intervention, Internet")) OR TS=("Intervention, Online")) OR TS=("social media*")) OR TS=("mobile")) OR TS=("web")) OR TS=("application")) OR TS=("App")) OR TS=("WeChat")) OR TS=("facebook")) OR TS=("twitter")) OR TS=("Telemedicine")) OR TS=("eHealth")) OR TS=("ehealth")) OR TS=("e-Health")) OR TS=("e-health")) OR TS=("tele-medicine")) OR TS=("Mobile Health")) OR TS=("Health, Mobile")) OR TS=("mHealth")) OR TS=("m-Health")) OR TS=("m-health")) OR TS=("telehealth")) OR TS=("tele-health")) OR TS=("Telerehabilitation")) OR TS=("Tele-rehabilitation*")) OR TS=("Remote Rehabilitation*")) OR TS=("Rehabilitation, Remote")) OR TS=("Virtual Rehabilitation*")) OR TS=("Rehabilitation, Virtual")) OR TS=("telecare")) OR TS=("tele-care")) OR TS=("telemonitoring")) OR TS=("tele-monitoring")) OR TS=("teleconsultation")) OR TS=("tele-consultation")) NOT (SILOID==("PPRN")) AND (TS=(exercise therapy) OR TS=(Remedial Exercise) OR TS=(Exercise, Remedial) OR TS=(Remedial Exercises) OR TS=(Therapy, Exercise) OR TS=(Exercise Therapies) OR TS=(Therapies, Exercise) OR TS=(Rehabilitation Exercise) OR TS=(Exercise, Rehabilitation) OR TS=(Exercises, Rehabilitation) OR TS=(Rehabilitation Exercises)) NOT (SILOID==("PPRN")) AND (TS=(osteoarthritis) OR TS=(Osteoarthritides) OR TS=(Osteoarthrosis) OR TS=(Osteoarthroses) OR TS=(Arthritis, Degenerative) OR TS=(Arthritides, Degenerative) OR TS=(Degenerative Arthritides) OR TS=(Degenerative Arthritis) OR TS=(Arthrosis) OR TS=(Arthroses) OR TS=(Osteoarthrosis Deformans)) NOT (SILOID==("PPRN"))

Search strategy in Cochrane library:

#1 MeSH descriptor: [Osteoarthritis] explode all trees #2 (Osteoarthritides or Osteoarthrosis or Osteoarthroses or Arthritis, Degenerative or Arthritides, Degenerative or Degenerative Arthritides or Degenerative Arthritis or Arthrosis or Arthroses or Osteoarthrosis Deformans):ti,ab,kw (Word variations have been searched)

#3 #1 OR #2

#4 MeSH descriptor: [Exercise Therapy] explode all trees

#5 (Exercises, Remedial or Rehabilitation Exercise or Remedial Exercise or Exercise, Rehabilitation or Exercises, Rehabilitation or Rehabilitation Exercises or Exercise Therapies or Remedial Exercises or Therapy, Exercise or Exercise, Remedial or Therapies, Exercise):ti,ab,kw (Word variations have been searched)

#6 #4 or #5

#7 MeSH descriptor: [Internet-Based Intervention] explode all trees

#8 (Intervention, Online or Intervention, Web-based or Internet Based Intervention or Internet Interventions or Online Interventions or Interventions, Online or Intervention, Internet or Web-based Interventions or Internet Intervention or Internet-Based Interventions or Interventions, Web-based or Online Intervention or Intervention, Internet-Based or Interventions, Internet-Based or Web-based Intervention or Web based Intervention or Interventions, Internet):ti,ab,kw (Word variations have been searched)

#9 #7 or #8

#10 #3 and #6
#11 #10 and #9

Search strategy in Embase

'arthritis, degenerative' OR 'arthritis, noninflammatory' OR 'arthrosis' OR 'degenerative arthritis' OR 'degenerative joint disease' OR 'noninflammatory arthritis' OR 'osteo-arthritis' OR 'osteo-arthrosis' OR 'osteoarthrosis' OR 'primary osteoarthritis' OR 'rheumatoid arthrosis' OR 'osteoarthritis'
AND
'corrective exercise' OR 'exercise movement techniques' OR 'exercise therapy' OR 'exercise treatment' OR 'kinesiotherapeutic intervention' OR 'kinesiotherapeutic method' OR 'kinesiotherapeutic procedure' OR 'kinesiotherapeutic technique' OR 'kinesiotherapeutical treatment' OR 'kinesitherapeutic exercises' OR 'kinesitherapeutic intervention' OR 'kinesitherapeutic method' OR 'kinesitherapeutic methodology' OR 'kinesitherapeutic procedure' OR 'kinesitherapeutic technique' OR 'kinesitherapeutic treatment' OR 'kinesitherapeutical treatment' OR 'kinesitherapy' OR 'SKTM (specialized kinesitherapeutic methodology)' OR 'specialised kinesitherapeutic methodology' OR 'specialized kinesitherapeutic methodology' OR 'therapeutic exercise' OR 'therapy, exercise' OR 'treatment, exercise' OR 'kinesiotherapy'
AND
'internet-based intervention' OR 'internet-intervention' OR 'online-based intervention' OR 'online-intervention' OR 'web intervention' OR 'web-based intervention'

Search strategy in EBSCO

AB Exercise Therapy OR Remedial Exercise OR Exercise, Remedial OR Exercises, Remedial OR Remedial Exercises OR Therapy, Exercise OR Exercise Therapies OR Therapies, Exercise OR Rehabilitation Exercise OR Exercise, Rehabilitation OR Exercises, Rehabilitation OR Rehabilitation Exercises
AND
AB Osteoarthritis OR Osteoarthritides OR Osteoarthrosis OR Osteoarthroses OR Arthritis, Degenerative OR Arthritides, Degenerative OR Degenerative Arthritides OR Degenerative Arthritis OR Arthrosis OR Arthroses OR Osteoarthrosis Deformans
AND
AB Internet-Based OR Internet Based Intervention OR Internet-Based Intervention OR Intervention, Internet-Based OR Web-based Intervention OR Intervention, Web-based OR Internet Intervention OR Intervention, Internet OR Intervention, Online OR social media OR mobile OR web OR application OR App OR WeChat OR facebook OR twitter OR Telemedicine OR eHealth OR ehealth OR e-Health OR e-health OR telemedicine OR tele-medicine OR Mobile Health OR Health, Mobile OR mHealth OR m-Health OR m-health OR telehealth OR tele-health OR Telerehabilitation OR Telerehabilitation OR Tele-rehabilitation OR Remote Rehabilitation OR Rehabilitation, Remote OR Virtual Rehabilitation OR Rehabilitation, Virtual OR telecare OR tele-care OR telemonitoring OR tele-monitoring OR teleconsultation OR

tele-consultation
